# Stroke awareness and response among university students in five Middle Eastern and North African countries: a multicenter cross-sectional study

**DOI:** 10.3389/fpubh.2026.1658947

**Published:** 2026-01-29

**Authors:** Asmaa Zakria Alnajjar, Moaz Elsayed Abouelmagd, Menna Marwan, Taleb Alsalloum, Abdelrahman Mady, Nagham Bushara, Yehia Nabil, Abdulrahman Krayim, Mahmoud Masoud, Maickel Abdelmeseh, Asma Daoud, Mohamed Alaa, Roaa Faisal, Mohamed Saad

**Affiliations:** 1 Medical Research Group of Egypt (MRGE), Negida Academy, Arlington, MA, United States; 2 Faculty of Medicine, Al-Azhar University, Gaza, Palestine; 3 Faculty of Medicine, Cairo University, Cairo, Egypt; 4 Faculty of Medicine, Port Said University, Port Said, Egypt; 5 Faculty of Medicine, University of Hama, Hama, Syria; 6 Faculty of Medicine, Al-Azhar University, Cairo, Egypt; 7 Faculty of Medicine, Zagazig University, Zagazig, Egypt; 8 Faculty of Medicine, Al-Azhar University, New-Damietta, Egypt; 9 Faculty of Medicine, Alexandria University, Alexandria, Egypt; 10 Faculty of Medicine, Ferhat Abbas University, Setif, Algeria; 11 Faculty of Medicine, Minia University, Minya, Egypt; 12 School of Medicine, Ahfad University for Women, Omdurman, Sudan; 13 Faculty of Medicine, Ain Shams University, Cairo, Egypt

**Keywords:** awareness, knowledge, MENA, stroke, university

## Abstract

**Background:**

Stroke is a major cause of mortality worldwide, including in the Middle East and North Africa (MENA) region. Enhancing stroke knowledge across society is crucial to improving outcomes. Limited data exist on stroke knowledge among university students in the MENA region. This study evaluates their stroke-related knowledge and behavior.

**Methods:**

We conducted a cross-sectional study to assess stroke knowledge and responses using the Stroke Knowledge Test (SKT) and Stroke Action Test (STAT) through an online questionnaire. Multivariate linear regression analyzed the relationship between sociodemographic factors and SKT/STAT scores.

**Results:**

A total of 1,169 participants (response rate 97.2%) completed the questionnaire, with a mean age of 21.67 years. Females comprised 68.8% of respondents. Participants were primarily from Egypt (41.9%), Palestine (19.2%), Algeria (15.7%), Jordan (11.6%), and Sudan (11.5%). The median SKT score was 11 (IQR: 9–14), with an overall accuracy of 68.17%. Gender, personal connection to stroke, and country significantly influenced SKT scores (*p* < 0.001). Egyptian and Algerian students scored highest (median 12, IQR: 9–14; *p* < 0.001). The STAT revealed a lower mean accuracy of 42.1%, with only 21.4% correctly identifying “sudden confusion” as a stroke emergency. Medical students achieved significantly higher SKT and STAT scores compared to nonmedical students and reported relying on educational curricula (75.5%) for information, while nonmedical students used social media, friends, and television.

**Conclusion:**

University students in the Arab world demonstrate relatively satisfactory stroke knowledge but insufficient practical response to stroke symptoms. Targeted medical educational initiatives and programs are needed to address this gap and improve stroke outcomes in Egypt.

## Introduction

Stroke is a medical condition characterized by the sudden disruption of blood flow to a specific part of the brain, resulting in brain cell damage or death (1). It represents a significant public health challenge globally, with significant implications for disability and mortality rates (2).

Methods for prevention and treatment have been effective in reducing the mortality rate of stroke in high-income countries over the past 50 years. Consequently, stroke has now become the fifth most prevalent cause of death in these countries, with a 42% decrease in mortality. Stroke continues to be the second leading cause of death (3–5).

A review of stroke epidemiology in the Middle East revealed a significant increase in the incidence rate of all strokes over the last decade, ranging from 22.7 to 250 per 100,000 people per year (5).

Factors such as age, having a family history of stroke, and living with chronic conditions such as diabetes, hypertension, and cardiovascular diseases are frequently found to increase the risk of stroke (6). Additionally, engaging in certain lifestyle behaviors, such as smoking and excessive alcohol consumption, is recognized as a stroke risk factor (7). Making lifestyle adjustments and managing modifiable risk factors can prevent 80% of stroke cases, emphasizing the significance of primary prevention (8).

The Arab region faces challenges in stroke awareness, as studies have revealed poor knowledge of stroke symptoms and risk factors among populations (9). According to studies conducted in Jordan and Egypt, 47 and 68.2%, respectively, of participants could not identify any of the stroke risk factors (10, 11).

This study aimed to evaluate the knowledge, attitudes, and practices (KAP) related to stroke among university students in Egypt, Palestine, Algeria, Jordan, and Sudan. University students play a critical role in stroke prevention, as they can significantly influence future health outcomes. Educating them about stroke risk factors and prevention empowers them to make informed decisions and serve as advocates for stroke prevention within their communities.

## Methods

### Study design and duration

We conducted a multinational, multicenter, descriptive, cross-sectional study with an analytic component between February 2024 and June 2024. The study included university students receiving medical and nonmedical education in five countries of the MENA region: Egypt, Algeria, Palestine, Jordan, and Sudan. Exclusion criteria included non-university students, participants outside the prespecified age which is between 17 and 28 years, and participants from countries other than the prespecified 5 countries. We followed the STROBE checklist.

### Sample size

For sample size calculation, we used the equation *N* = *z*^2^^*^ *p* ^*^ *q*/*d*^2^, where *N* is the sample size required from each country, “*z*” is the reliability coefficient set at 1.96 to obtain a 95% confidence interval, *p* (expected frequency) = 50% to obtain the largest sample size, “*q*” (1–*p*) = 50% and *d* represents the margin of error set at 8% (12). Using this formula, we calculated a sample size of 135 participants per country, resulting in a minimum total of 675 participants.

### Sampling and data collection approach

Participants were recruited by convenience sampling from the selected countries. The data were collected for 1 month, from 6th April to 6th May. Our target population was university students receiving medical and nonmedical education, including those studying Medicine, Engineering, Science, Arts, Education, Dentistry, Business, and others. The students completed the questionnaire using Google Forms. We sent the questionnaire to students in all academic years through official channels on the Telegram app and other social media platforms where students are added by the student council. This strategy targeted students directly, restricting access to those without institutional ties. Each author was responsible for collecting the data from their countries. Additionally, collaborators were recruited from Jordan to help with the data collection. The authors trained them on how to approach students online.

### Questionnaire

We based our questionnaire on the Stroke Knowledge Test (SKT) (13, 14) and the Stroke Action Test (STAT) (15), which have validated Arabic versions. These tests assess participants' stroke knowledge and practices. Before distribution, we piloted the survey on 10 subjects to refine it. The final version of the questionnaire consisted of four sections as follows:

The first section focused on sociodemographic traits. The registered data included age, sex, county, living area, college education, income level, family history of stroke, and knowledge of someone with stroke.

The second part of our questionnaire was the Stroke Knowledge Test (SKT), a 20-item questionnaire developed by Sullivan and Dunton (13). The goal of the test was to assess the nature of stroke, its associated risk factors, and its consequences. Participants completed all 20 items, comprising true/false and multiple-choice questions covering stroke definition, common symptoms (e.g., sudden weakness and speech difficulty), major risk factors (e.g., hypertension and smoking), and appropriate immediate actions. The proportion of correct responses varied across items, with higher accuracy for the general definition of stroke and well-known risk factors, and lower accuracy for specific symptoms and less commonly recognized risk factors. A cut-off point for good knowledge was defined as correctly answering at least 50% of the items (i.e., 10 out of 20 questions) (13). The tool has demonstrated good internal consistency, test-retest reliability, and construct validity, with a Cronbach coefficient of 0.65 (13, 14). The instrument was translated into Arabic and validated in a previous study (10), where face and content validity were established with a Cronbach's alpha of 0.68. We obtained permission to use the tool directly from the developers. To ensure its reliability in our specific study population and context, we re-assessed the internal consistency, which yielded a Cronbach's alpha of 0.914.

The third part of our questionnaire was the Stroke Action Test (STAT), which is a 28-item written instrument developed by Billings-Gagliardi and Mazor (15). The reliability of the 28-item test was good, as determined by a Cronbach's alpha of 0.83 (15). The Arabic version of the questionnaire was validated in a prior study (16), where reliability was established with a Cronbach's alpha of 0.923. We obtained permission to use both versions from the developers. To confirm the tool's applicability to our sample, we re-evaluated its reliability, which resulted in a Cronbach's alpha of 0.877.

The last section of our questionnaire concerned the respondents' sources of information about stroke (TV, health care professionals, Advertisement and media, friends and relatives, or newspapers).

### Data analysis

The data were analyzed using Jamovi version 2.3.18 (17). For descriptive statistics, categorical and ordinal data are presented as frequencies. Normally distributed continuous data are presented as the mean and standard deviation (SD), while skewed data are presented as the median and interquartile range (IQR). The normality of the distributions was assessed by the Shapiro–Wilk test, for which a *p*-value < 0.5 rejects the assumption of a normal distribution.

A reliability test was performed for each of the questionnaires using Cronbach's alpha to determine internal consistency. The Mann–Whitney *U* test and Kruskal–Wallis *H* test were used to assess the association between each continuous independent variable (knowledge: SKT score; practice: STAT score) and the sociodemographic variables. Multivariate linear regression models were constructed to examine factors associated with SKT and STAT scores as dependent variables, taking all variables that had a *p*-value < 0.25 (18) in the bivariate analysis as independent variables. A two-sided *p* < 0.05 was considered to indicate statistical significance.

### Ethical considerations

This study was conducted in accordance with the ethical principles outlined in the Declaration of Helsinki. Ethical approvals were obtained from the Institutional Research Boards of the Faculty of Medicine, Kasr Al-Ainy, Cairo University (Approval No. 382-2024), Al-Azhar University in Gaza, Palestine (Approval No. 154-2024), and the Faculty of Medicine, Ferhat Abbas University in Setif, Algeria (Approval No. 85-2024). All participants provided electronic informed consent before participating in the online survey. Participation was voluntary, anonymous, and uncompensated. Respondents were informed that they could complete the questionnaire at their convenience and withdraw at any time without any consequences. Data confidentiality was strictly maintained through anonymization and secure digital storage, accessible only to the research team. The study posed no physical or psychological risk to participants.

## Results

A total of 1,169 participants (response rate 97.2%) met the inclusion criteria and were included in the analysis. The sociodemographic characteristics of the study participants are summarized in Table 1. The mean age was 21.67 years, and 68.8% (*n* = 804) of respondents were female. Participants were primarily from Egypt (41.9%, *n* = 490), Palestine (19.2%, *n* = 225), Algeria (15.7%, *n* = 183), Jordan (11.6%, *n* = 136), and Sudan (11.5%, *n* = 135). Most participants lived in urban areas (75.4%, *n* = 882), were enrolled in medical programs (70.5%, *n* = 824), and reported a medium income level (87.0%, *n* = 1,017). A majority (88.8%, *n* = 1,038) reported no family history of stroke, while 37.0% (*n* = 433) knew someone who had experienced a stroke.

**Table 1:** Sociodemographic characteristics of study participants.

**Characteristic**	**Category/Statistic**	**Egypt (*n* = 490)**	**Jordan (*n* = 136)**	**Palestine (*n* = 225)**	**Sudan (*n* = 135)**	**Total (*n* = 1,169)**	***p*-value**
**Age**							< 0.001^a^
Mean (SD)	22.98 (1.84)	21.80 (1.79)	20.69 (1.61)	20.81 (1.71)	21.80 (2.69)	21.67 (2.02)	
**Gender**, ***n*** **(%)**							< 0.001^b^
Male	18 (9.8%)	225 (45.9%)	35 (25.7%)	45 (20.0%)	42 (31.1%)	365 (31.2%)	
Female	165 (90.2%)	265 (54.1%)	101 (74.3%)	180 (80.0%)	93 (68.9%)	804 (68.8%)	
**Living area**, ***n*** **(%)**							0.011^c^
Urban	151 (82.5%)	315 (64.3%)	124 (91.2%)	172 (76.4%)	120 (88.9%)	882 (75.4%)	
Rural	32 (17.5%)	175 (35.7%)	12 (8.8%)	53 (23.6%)	15 (11.1%)	287 (24.6%)	
**Education**, ***n*** **(%)**							0.048^c^
Medical	152 (83.1%)	376 (76.7%)	88 (64.7%)	101 (44.9%)	107 (79.3%)	824 (70.5%)	
Non-medical	31 (16.9%)	114 (23.3%)	48 (35.3%)	124 (55.1%)	28 (20.7%)	345 (29.5%)	
**Income level**, ***n*** **(%)**							0.048^c^
Low	16 (8.7%)	30 (6.1%)	8 (5.9%)	27 (12.0%)	9 (6.7%)	90 (7.7%)	
Medium	163 (89.1%)	440 (89.8%)	116 (85.3%)	185 (82.2%)	113 (83.7%)	1,017 (87.0%)	
High	4 (2.2%)	20 (4.1%)	12 (8.8%)	13 (5.8%)	13 (9.6%)	62 (5.3%)	
**Family history of stroke**, ***n*** **(%)**							0.048^c^
No	157 (85.8%)	432 (88.2%)	120 (88.2%)	212 (94.2%)	117 (86.7%)	1,038 (88.8%)	
Yes	26 (14.2%)	58 (11.8%)	16 (11.8%)	13 (5.8%)	18 (13.3%)	131 (11.2%)	
**Know someone with stroke**, ***n*** **(%)**							< 0.001^c^
No	82 (44.8%)	325 (66.3%)	96 (70.6%)	155 (68.9%)	78 (57.8%)	736 (63.0%)	
Yes	101 (55.2%)	165 (33.7%)	40 (29.4%)	70 (31.1%)	57 (42.2%)	433 (37.0%)	
SKT score (median, IQR)	12 (4)	12 (5)	11 (7)	10 (5)	11 (5)	11 (5)	< 0.001^a^

N, number of students per group; SD, standard deviation.

^a^*P*-value using Shapiro–Wilk test.

^b^*P*-value using chi-square chi test.

^c^*P*-value using student's test.

### Knowledge of stroke

The overall median score on the Stroke Knowledge Test (SKT) was 11 (IQR: 9–14), corresponding to an accuracy rate of 68.17%. Respondents from Egypt and Algeria achieved the highest median scores (12 each), while those from Palestine had the lowest (median 10). In bivariate analysis, SKT scores were significantly associated with gender, knowing someone with stroke, and country (all *p* < 0.05), but not with education type, living area, family history of stroke, or income level (Table 2).

**Table 2:** Bivariate analysis of factors associated with SKT and STAT scores.

**Variable**	**SKT score [median (IQR)]**	***p*-value (SKT)**	**STAT score [median (IQR)]**	***p*-value (STAT)**
**Education type**		0.059		< 0.001
Medical	12 (5)		12 (11)	
Non-medical	11 (5)		9 (8)	
**Country**		< 0.001		0.016
Algeria	12 (4)		12 (11.5)	
Egypt	12 (5)		12 (9)	
Jordan	11 (7)		10 (11)	
Palestine	10 (5)		10 (10)	
Sudan	11 (5)		10 (10)	
**Living area**		0.978		0.186
Rural	11 (5)		12 (10)	
Urban	11 (5)		11 (11)	
**Gender**		0.034		0.195
Male	12 (5)		11 (11)	
Female	11 (6)		11 (10)	
**Family history of stroke**		0.360		< 0.001
Yes	12 (5.5)		14 (9.5)	
No	11 (5)		11 (10)	
**Know someone with stroke**		0.048		< 0.001
Yes	12 (5)		13 (10)	
No	11 (6)		10 (10)	
**Income level**		0.202		0.002
High	11 (4)		14 (10)	
Medium	12 (5)		11 (11)	
Low	10.5 (5)		10 (8)	

IQR, inter-quartile range; SKT, stroke knowledge test; STAT, stroke action test.

*p*-Values derived from Mann–Whitney U or Kruskal–Wallis tests.

In multivariate linear regression analysis **(using Egypt as the reference category)**, male gender (Beta = 0.599, *p* = 0.016) was associated with higher SKT scores, while knowing someone with stroke (Beta = −0.589, *p* = 0.013) and being from Jordan (Beta = −1.427, *p* < 0.001), Palestine (Beta = −1.052, *p* < 0.001), or Sudan (Beta = −1.139, *p* = 0.002) were associated with lower scores (Table 3).

**Table 3:** Multivariate linear regression analysis of factors associated with SKT and STAT scores.

**Variable (reference)**	**Beta (SE)**	**95% CI**	***p*-value**
**SKT model**			
Gender (female ref.)	0.599 (0.247)	0.114–1.084	0.016
Knows someone with stroke (yes ref.)	−0.589 (0.237)	−1.054 to −0.124	0.013
**Country (Egypt ref.)**			
Jordan	−1.427 (0.375)	−2.162 to −0.692	< 0.001
Palestine	−1.052 (0.311)	−1.662 to −0.442	< 0.001
Sudan	−1.139 (0.376)	−1.876 to −0.402	0.002
**STAT model**			
Age	0.432 (0.090)	0.255–0.609	< 0.001
Education (medical ref.)	−1.535 (0.403)	−2.325 to −0.745	< 0.001
Knows someone with stroke (yes ref.)	−1.176 (0.378)	−1.917 to −0.435	0.002
**Income (high ref.)**			
Low	−2.908 (1.011)	−4.890 to −0.926	0.004
Medium	−2.657 (0.799)	−4.223 to −1.091	< 0.001
Family history of stroke (yes ref.)	−2.230 (0.579)	−3.365 to −1.095	< 0.001
**Country (Egypt ref.)**			
Jordan	−1.731 (0.603)	−2.913 to −0.549	0.004
Sudan	−1.369 (0.605)	−2.555 to −0.183	0.024

SE, standard error; CI, confidence interval; SKT, stroke knowledge test; STAT, stroke treatment awareness test.

Reference categories are indicated in parentheses.

### Response to stroke

The mean accuracy on the Stroke Action Test (STAT) was 42.1%, with only 21.4% (*n* = 250) of participants correctly identifying “sudden confusion” as a stroke emergency. The most recognized symptoms prompting emergency calls were muscle weakness in one arm (78.7%, *n* = 920) and inability to talk (64.0%, *n* = 748). Factors significantly associated with higher STAT scores in bivariate analysis included medical education, country, family history of stroke, and knowing someone with stroke (Table 2).

Multivariate regression **(using Egypt as reference)** identified older age (Beta = 0.432, *p* < 0.001), medical education (Beta = −1.535 for non-medical, *p* < 0.001), having a family history of stroke (Beta = −2.230 for no history, *p* < 0.001), and higher income level as positive predictors. Participants from Jordan (Beta = −1.731, *p* = 0.004) and Sudan (Beta = −1.369, *p* = 0.024) scored significantly lower than those from Egypt (Table 3).

### Sources of information

**Figure 1:**
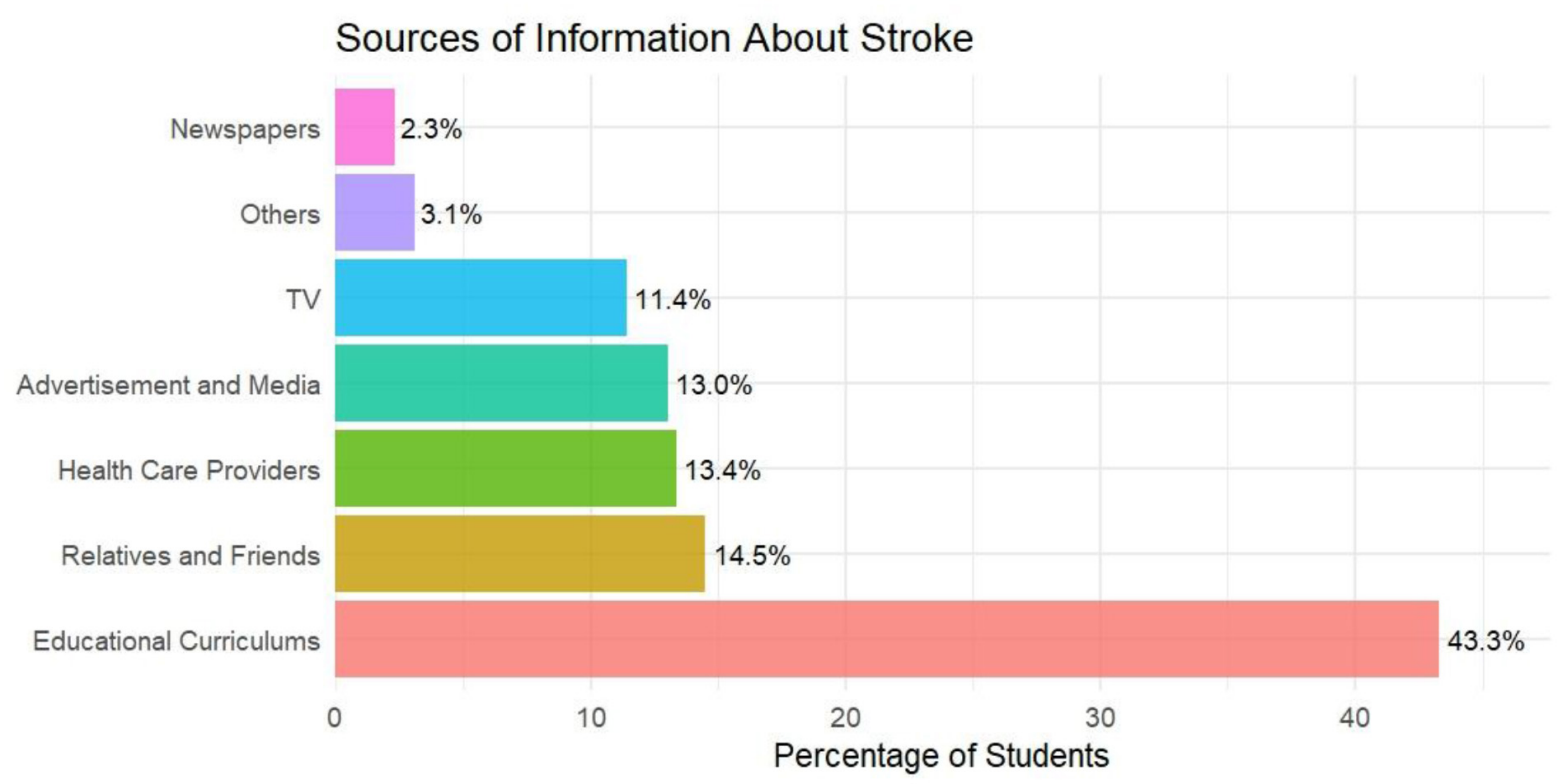
Sources of information about stroke incidence.

The primary source of stroke information was educational curricula (43.3%, *n* = 506), followed by relatives and friends (14.5%, *n* = 170) and healthcare providers (13.4%, *n* = 157). Only 2.3% (*n* = 27) cited newspapers. Medical students relied predominantly on educational curricula (75.5%), whereas non-medical students used more diverse sources such as advertisement/media (34.0%), relatives/friends (34.8%), and television (23.7%; Figure 1).

## Discussion

### Knowledge of stroke

The collective stroke awareness among university students across the region was high compared to a recent study in Saudi Arabia, where only 45.5% of participants demonstrated satisfactory awareness (19). In contrast, a study in Jordan revealed a more positive outcome, with 87.3% of the population aware of at least one stroke sign or symptom (20). This variability in awareness levels is evident across different national and educational contexts. For instance, studies among university students in Pakistan and Sudan reported high recognition of stroke as a brain disease (94.9 and 93.2%, respectively), though comprehensive knowledge remained suboptimal, with only 40.2% in Sudan identifying all correct answers about stroke (21, 22). Long-term trends from the United States show public knowledge of stroke warning signs and risk factors improved between 1995 and 2021, but gains have stagnated or declined since 2011, particularly among women and those with lower educational attainment (23). This underscores the challenge of sustaining awareness over time, even in high-resource settings.

Overall, the findings highlight a substantial gap in comprehensive stroke knowledge, a pattern that has been consistently reported across diverse populations. Only a minority of individuals demonstrate an integrated understanding of stroke risk factors, warning symptoms, and the need for urgent medical action, with one population-based study reporting adequate combined knowledge in just over one-third of participants (24). Across studies, higher educational attainment, female sex, urban residence, and personal or family history of stroke or cardiovascular disease have repeatedly emerged as key predictors of better stroke awareness (24, 25). Recognition of stroke symptoms appears to be moderate at best, with individuals typically identifying only a subset of established warning signs. Improved symptom recognition has been consistently linked to higher education levels, numeracy skills, and prior exposure to cardiovascular disease, either personally or within the family (25).

Regarding stroke risk factors, the majority of our participants correctly identified the primary risk factor, with Egypt showing the highest percentage (22.1%). These results were consistent with the Saudi Arabia study, in which 32.8% demonstrated good knowledge of primary stroke risk factors (26). However, another study in Egypt found that 68.6% lacked awareness of stroke risk factors, possibly due to disparities between rural and urban populations, with urban areas displaying higher awareness (11). Hypertension is frequently identified as the most recognized risk factor across various settings, as seen in Sudan (90.2%), Pakistan (58.4%), and mirrored in our findings (21, 22). This pattern aligns with observations from Northwest India, where higher education was strongly associated with better knowledge of stroke warning symptoms and risk factors among patients and their relatives (27). However, recognition patterns can vary; for example, a Polish study found old age to be the most frequently cited risk factor (55.3%), while hypertension was recognized by only 29.2% of respondents (24).

### Response to stroke

The data suggest no major differences among countries, except for Egypt and Jordan, where participants had better reactions to stroke symptoms. In our study, the majority did not seek emergency medical services (EMS) for stroke emergencies. Similar findings were observed in Lebanon, where 41.5% of respondents did not choose to call EMS for more than six stroke symptoms (16). A common theme across studies is the support for hospitalization as the immediate response, though significant gaps remain. In Quetta, 88.2% of students supported hospitalization, while 5.1% preferred traditional healing practices (21). Similarly, in Sudan, 86.3% would transport a suspected stroke patient to the hospital (22). Post-campaign data from Malaysia showed significant improvement, with 99.4% indicating they would call an ambulance after exposure to a stroke awareness campaign (28). A lack of awareness about stroke symptoms and appropriate response has been shown to result in significant delays in seeking medical help, ultimately reducing survival chances.

A disconnect between the intent to act and the ability to recognize stroke symptoms is commonly reported. For instance, while 82.6% of participants in Poland indicated they would call EMS for a suspected stroke, 42.4% could not name a single stroke symptom (24). This gap is echoed in China, where 75.9% would call an ambulance, but the second most common response was to “advise seeing a doctor” (58.8%) rather than seeking immediate emergency consultation (34.1%) (25). Females in our sample showed better responses to stroke, consistent with previous studies (15, 16, 20) and with findings from Poland and China where female gender was associated with better stroke knowledge and a higher likelihood of appropriate action (24, 25). This association is further supported by a study in Sudan, where females had significantly higher odds of identifying at least one stroke risk factor compared to males (22).

The symptoms most recognized as reasons to call EMS were muscle weakness in one arm and difficulty speaking clearly, aligning with previous studies in Lebanon (16) and Jordan (20) and the core FAST (Face, Arm, Speech, Time) mnemonic. Recognition of FAST signs can be significantly improved through targeted campaigns, as demonstrated in Malaysia where recognition increased by 80% following an intervention (28). In our study, visual disturbance in one eye was also identified as a stroke indicator, differing from some previous studies (15, 20, 29). Participants did not widely recognize dizziness or comprehension difficulties as stroke symptoms, possibly due to medical terminology or unclear survey descriptions (15). Less common symptoms like sudden confusion, swallowing difficulties, and one-sided blindness are often poorly recognized by the public, as seen in China where fewer than 25% of respondents identified them, suggesting public education often fails to cover the full spectrum of stroke presentations (25).

### System-level factors and prehospital delays

In settings with well-developed EMS systems, such as Finland, correct stroke identification by dispatchers and efficient first response unit deployment have been shown to reduce on-scene time (30). However, public knowledge gains are not guaranteed to persist; longitudinal data from the U.S. indicate improvements in risk factor knowledge can decline over time, especially among those with lower educational attainment (23). The critical link between public awareness and timely treatment is emphasized by studies noting that prompt hospital admission is essential for effective thrombolysis (24). Innovations in prehospital care, such as specialized stroke ambulances with telemedicine and CT scanners, have reduced alarm-to-treatment times and increased thrombolysis rates, bringing treatment into the critical “golden hour” (31). Such system-level efforts highlight the importance of integrating public education with optimized EMS protocols to minimize delays. Access to specialized stroke units significantly reduces mortality and improves independence, particularly in underserved rural areas (32).

Our study showed no association between stroke knowledge, as measured by SKT scores, and stroke attitudes, as measured by STAT scores, consistent with previous studies (16, 33). This disconnect indicates that knowledge alone may not translate to appropriate action, a challenge noted in evaluations of mass media campaigns (34). In Malaysia, despite improved knowledge and action after a campaign, nearly half of respondents still considered non-emergency help, indicating deeper behavioral and cultural factors need addressing (28). The influence of health literacy is further highlighted by a Chinese study which identified numeracy as an independent factor influencing symptom recognition, suggesting health communication must be accessible to those with lower numerical literacy (25).

### Source of information

In our study, 75.5% of medical students received stroke information through educational curricula, a reliable source. Medical students dedicate more time to studying stroke through credible sources, allowing them to recognize symptoms and respond appropriately. This aligns with findings where high stroke knowledge is observed among educated samples, such as university students in Pakistan (21). Non-medical students often rely on less trustworthy sources such as advertisements, media, acquaintances, and television. The reliance on digital sources is prominent; in Sudan, the primary sources were the internet and social media (79.3%), followed by healthcare professionals (60%) (22), a pattern also observed post-campaign in Malaysia (28). Non-medical students, as digital natives, frequently turn to online platforms but may lack critical appraisal skills, a challenge noted in studies of online health information-seeking behaviors (28).

In Ismailia, personal encounters with someone who experienced a stroke were the primary knowledge sources (63.2%), with family members playing a significant role (52.4%) (11). Advertisements and media played a minor role, consistent with findings from Cairo University Hospitals, where mass media represented only 10.9% of participants (35). These findings emphasize the need to leverage social media platforms for stroke awareness, similar to the multimedia approach used in the Act FAST campaign in the U.S (36). Television remains a main source of stroke information in some contexts, such as Poland (58.7%), yet studies suggest TV campaigns alone may be insufficient to build adequate knowledge, indicating a need for more detailed and emotionally impactful messaging (24). The effectiveness of health materials may also depend on public numeracy, as highlighted by a Chinese study noting that most health materials contain numerical data (25).

### Clinical implications

Timely recognition of stroke symptoms is crucial for initiating immediate medical intervention. Assessing knowledge of stroke symptoms enables healthcare providers to identify gaps and implement targeted educational programs. Media-driven initiatives, like Malaysia's ResQ Stroke Campaign, demonstrate that public knowledge, recognition of FAST signs, and healthcare-seeking behavior can be significantly improved. Tailored interventions are needed in regions where awareness remains suboptimal, such as Sudan, targeting individuals with inadequate stroke literacy. General awareness campaigns may be insufficient to improve thrombolysis rates or promote healthy behaviors; recommendations from Poland suggest more impactful education leveraging emotional connections, such as referencing relatives' experiences or using virtual patient presentations. Similarly, promoting simple detection tools like the FAST test is recommended to bridge the gap between symptom recognition and action, especially for those with lower numeracy.

### Limitations and strengths

This study has both strengths and weaknesses. It is the first to gather evidence from five MENA countries, offering a comprehensive evaluation of stroke knowledge levels and responses. However, using a specific sample of university students limits generalizability to other regions and broader populations, as noted in Pakistan and Sudan. Conducting a Google Form survey may introduce selection bias by excluding students with limited access to technology. The use of closed-ended questions could overestimate stroke knowledge, a methodological concern echoed in the Polish study, which used open-ended questions and found knowledge was lower when recall was required. Self-reporting bias is possible, and the cross-sectional design limits the ability to establish causality or track changes over time. Students were selected due to accessibility and their role as an educated young group critical for future health promotion. Nevertheless, findings may not fully generalize to the wider population, as students can differ in socioeconomic, educational, and health-related aspects. Despite these limitations, the study emphasizes the importance of targeted educational interventions and collaboration with educational institutions to strengthen stroke awareness and prevention among university students in the Arab world.

## Conclusion

Our research findings demonstrate that university students in the Arab world generally possess satisfactory knowledge about stroke. However, their practical response to stroke situations was found to be insufficient. This highlights the importance of implementing focused interventions to address the gap between knowledge and effective response. To improve stroke outcomes and alleviate the associated burden, it is essential to enhance response measures such as early symptom recognition and prompt medical attention. Moving forward, it is crucial to prioritize initiatives that raise awareness, reduce response times, and provide the necessary resources to ensure optimal stroke care in the Arab world.

## Data Availability

The original contributions presented in the study are included in the article/supplementary material, further inquiries can be directed to the corresponding authors.
